# Metabolites: a converging node of host and microbe to explain meta-organism

**DOI:** 10.3389/fmicb.2024.1337368

**Published:** 2024-03-05

**Authors:** Nabarun Chakraborty

**Affiliations:** Medical Readiness Systems Biology, CMPN, WRAIR, Silver Spring, MD, United States

**Keywords:** metabolite, microbiome, metagenomics, holobiont, fecal microbes, gut brain axis, microbial diversity, small molecules

## Abstract

Meta-organisms encompassing the host and resident microbiota play a significant role in combatting diseases and responding to stress. Hence, there is growing traction to build a knowledge base about this ecosystem, particularly to characterize the bidirectional relationship between the host and microbiota. In this context, metabolomics has emerged as the major converging node of this entire ecosystem. Systematic comprehension of this resourceful omics component can elucidate the organism-specific response trajectory and the communication grid across the ecosystem embodying meta-organisms. Translating this knowledge into designing nutraceuticals and next-generation therapy are ongoing. Its major hindrance is a significant knowledge gap about the underlying mechanisms maintaining a delicate balance within this ecosystem. To bridge this knowledge gap, a holistic picture of the available information has been presented with a primary focus on the microbiota-metabolite relationship dynamics. The central theme of this article is the gut-brain axis and the participating microbial metabolites that impact cerebral functions.

## Introduction

The total number of resident microbiota, or the “collection of microorganisms living in or on the human body,” (Merriam-Webster, n.d.) marginally exceeds the number of human cells (Sender et al., 2016). A 70 kg adult male is estimated to have 39 trillion bacteria that live with 30 trillion human cells, which includes both nucleated and non-nucleated cells, e.g., red blood cells (Sender et al., 2016). The ratio between bacterial and host cells varies from 1.3 in adult males to 2.2 in adult females (Sender et al., 2016). The higher microbial cellular load in adult females is attributed to a unique and complex ecosystem of microbes colonized in the female genital tract (Punzon-Jimenez and Labarta, 2021) and vagina (Fu et al., 2020; Srinivasan et al., 2022), and this ecosystem alters with the menstruation cycle (Krog et al., 2022) and during pregnancy (Punzon-Jimenez and Labarta, 2021). Hence, it is essential to consider this diverse microbial ecosystem across the genders (Auriemma et al., 2021) to make any inference in this field of study.

The endogenous microbiota of the human body is mostly concentrated inside the intestine. The gut microbial community is predominantly enriched by bacteria (10^9^–10^11^ cells/g) (Sender et al., 2016; Cani, 2018) and archaea (10^8^–10^10^ cells/g) (Kim et al., 2020). Another major microbe that colonizes the intestinal lumen is the virus, an intracellular parasite, and the bacteriophage is a major viral species that controls the bacterial diversity in the host (Scanlan, 2017). The bacteria outnumber viruses by approximately 10 to 1, as there are 10^9^–10^10^ virus-like particles (VLP) per gram of human feces (Shkoporov and Hill, 2019). While the human genome contains approximately 20,000 genes, the hologenome, a combination of the host and resident microbes, contains over 33 million genes (Lloyd-Price et al., 2016). Ninety-three percent of these genes belong to bacteria (Sender et al., 2016), while viruses claim the second largest share, e.g., 5.8% of total DNA (Arumugam et al., 2011). Together, this gut microbiota fosters a balanced ecosystem with its host, and this interactive milieu is the core feature of the meta-organism (Theis et al., 2016) or holobiont concept (Simon et al., 2019). In theory, the meta-organism or holobiont concept is about studying the holistic host-microbiota interactive sphere that includes biology, ecology, and evolution of both host and resident-microbiota. For the present purpose, we will focus on the biology of meta-organisms.

The functional microbiome (Lam et al., 2015; Berg et al., 2020) is constituted by different omics layers linked to the microbe, namely metagenomics, metatranscriptomics, metaproteomics, and meta-metabolomics, often called metabolomics (Zhang et al., 2019; Zou et al., 2019; Salvato et al., 2021). One of the significant operations of a functional microbiome is to maintain a robust crosstalk between the resident microbe and the host’s peripheral tissues, such as the heart, lungs, kidney, and brain. The gut-brain axis, possibly the most studied subject in this context, embodies the bidirectional communication between the host’s brain and gut commensals that control several brain functions, such as neuroinflammation, neurodegeneration, neurotoxicity, and behavioral, emotional, and memory constructs (Mayer et al., 2015; Cryan et al., 2020).

Eubiosis, or the *balanced* abundance profile of resident microbiota (Iebba et al., 2016; Al-Rashidi, 2022) fosters a symbiotic relationship with the host when the resources available to the host become systematically shared with its resident microbiota. Eubiotic microbial composition controls inflammation and maintains energy homeostasis and a robust gut-brain axis (Lloyd-Price et al., 2016). In contrast, stressful conditions, such as changes in lifestyle or challenges from foreign elements, elevate the host’s demand for the resources, eventually forcing the host and microbiota to compete for resources from a shared pool. Consequently, as the microbial diversity alters, the adaptive and facultative microbiota proliferate, and the overall ecosystem shifts into dysbiosis that could disrupt the host-microbial communication, including the gut-brain axis (Chen and Devaraj, 2018; Dabke et al., 2019; Louis-Jean and Martirosyan, 2019; Cryan et al., 2020; Xu et al., 2020).

**Figure 1:**
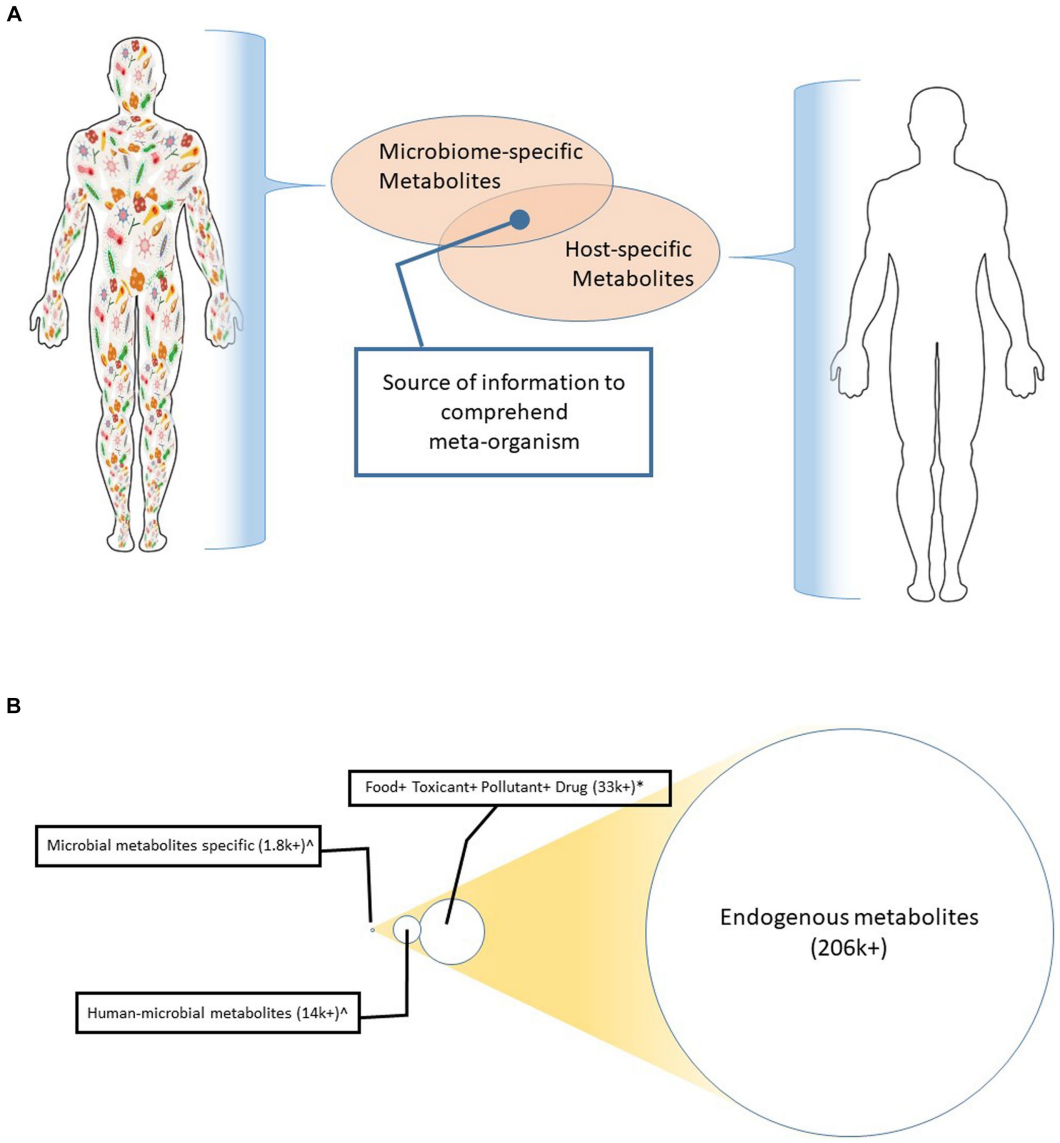
**(A)** Metabolite as a converging node of multiple kingdoms. This illustration depicts that the host (e.g., human in this case) produces a metabolite cluster. Likewise, the resident microbiota generates another set of metabolite clusters. Characterization of the functional interaction of these two clusters can inform the underlying mechanisms that control the meta-organism. **(B)** A comparative number of the metabolites generated from humans, the environment, and those microorganisms that colonize in or on humans. The circles are drawn in scale to give an eye estimation of the differences in their abundances.

Metabolites are the key information hub of meta-organisms since the host-microbiota communication grid, including the gut-brain axis, is built upon the exchange of metabolites (Ramautar et al., 2013; Wishart, 2019; Wachsmuth et al., 2022). Being the intermediate and derivatives of the biological networks in host cells and microorganisms alike, metabolites appear to be the converging node of the ecosystem (Krautkramer et al., 2021). Illustrating this concept, Figure 1A depicts metabolites as the major node of the crosstalk between the host and its resident microbiota. Figure 1B shows the sizes of various metabolite superfamilies; these metabolite superfamilies are linked to the host, environment, resident microbiome, and their interphases. Microbial metabolites are the smallest in number (Krautkramer et al., 2021).

Rather than curating citations via preset exclusion-inclusion criteria, the present review article selected citations driven by its hypothesis and crafted the review in the following order. The first chapter briefly describes metabolomics, focusing on the available detection tools and pertinent databases to illuminate the metabolites’ association with other omics components. The second chapter describes the resident microbiome, its diversity profile, and how this ecosystem controls the host’s health. The third chapter focuses on the microbial metabolites, e.g., those synthesized by commensals colonized in or on the host. The fourth chapter discusses the gut-brain axis; the potential roles of metabolites and microbiome in supporting this communication framework, and the pathogenesis of the diseases linked to the central nervous system (CNS) are discussed in adjunct. The concluding section highlights the knowledge gap in comprehending meta-organisms and suggests future applications in healthcare and beyond. Significant terms used in this review article are defined in Box 1.

## Metabolites: current perspective

Metabolites are the substrate, intermediate, and end product of the biological activities at the cells, tissues, or organ levels (Ramautar et al., 2013; Wishart, 2019). Metabolites are the typical downstream products of the host’s genomic, epigenomic, and proteomic activities. Present capabilities can detect more than 200,000 endogenous metabolites linked to ~1,900 metabolic enzymes encoded in the human genome (Kanehisa and Goto, 2000). In comparison, microbes produce approximately 16,000 metabolites, of which nearly 1,800 are exclusively produced by the microbe community, and the rest are produced by both humans and microbes. In addition, many metabolites are linked to the environment. For instance, ~32,000, ~900, and ~160 metabolites are associated with diet, toxins, and drugs, respectively (Metabolite Statistics, n.d.). Approximately 1 to 3 million xenobiotic metabolites or synthetic products represent drugs, cosmetics, food supplements, pollutants, etc. (Idle and Gonzalez, 2007; Johnson et al., 2012), and many overlap with those linked to the environment. A subset of these metabolites is classified as toxic substances and foreign stimulants to meta-organisms (Gonzalez-Sanchez and DeNicola, 2021).

Emerging knowledge has associated the metabolites’ roles with many biological functions, such as disease pathogenesis (Mamas et al., 2011), immune modulations (Levy et al., 2016), and trans-organ communications (Levy et al., 2016; Frezza, 2017). Indeed, the alterations of biochemical activities due to physiological and psychological stress, exposure to external stimulants, or shifts in diet or lifestyle changes alter the metabolite expressions (Liu et al., 2021) and ultimately influence the overall health of the meta-organism (Milovic et al., 2000; Amaral et al., 2009). Therefore, metabolomics holds time-sensitive information on the molecular activities that co-occur across multiple organisms comprising the host, its resident microbiota, and the overarching environment.

The technical capability of detecting the metabolite has reached a high standard of resolution and robustness in recent years due to the development of cutting-edge tools and highly enriched databases. Table 1 lists the leading tools available to detect and characterize metabolites. Mass spectroscopy-based tools are most popular due to their high throughput and highly sensitive detection capabilities (Dührkop et al., 2021). Recent technological developments in the miniaturization of spectrophotometers have revolutionized their applicability since these tools have become increasingly portable, affordable, and easy to operate in austere conditions (Alseekh et al., 2021). There are supplementary tools available that can further enrich our knowledge of metabolites. For instance, the nuclear magnetic resonance (NMR) spectroscopy can divulge the metabolites’ structures (Wishart et al., 2022). The capability of Raman spectroscopy in detecting metabolites with spatial resolution has recently been adapted to study single-cell metabolomics (Berry and Loy, 2018). A low throughput option, such as histochemistry, presents a unique scope to detect the spatial enrichment of targeted metabolites. The spatial information could be vital in mapping metabolites to the central nervous system (CNS). It can illuminate the differential metabolomic expressions across the blood-brain barrier (BBB). Speculatively, this knowledge can help to understand the roles of metabolites in the gut-brain axis (Mayer et al., 2015).

**Table 1:** Comparative description of the instruments/tools to measure metabolites.

Instrument/Tool	Throughput	Sensitivity	Specificity	Unique features/limitations
Instruments using mass spectrometry (MS), such as liquid chromatography (LC)-MS, gas chromatography (GC)-MS, MALDI, etc.	High; however, low throughput targeted assays could be accomplished	High	Depends on the tool selection. For instance, the LC-MS tool is highly proficient in measuring volatile compounds, whereas GC-MS tools are excellent platforms for detecting polar/non-polar metabolites	Sample identification depends on the maturity of the database, which has been evolving continuously. Also, this technique cannot provide the structural information of the molecule
Nuclear magnetic resonance (NMR) spectroscopy	High, but lower than MS-based tools	High, but lower than MS-based tools	Highly specific, possibly more than MS-based tools	Uniquely capable of detecting the structure of metabolite
Raman spectroscopy	Moderate	Moderate	Highly specific	Uniquely capable of detecting with spatial resolution and structural identification without destroying the samples so that the samples could be repurposed

Development of pertinent databases remains an ongoing effort since the technological capabilities in identifying the metabolites remain a limiting step for constructing the databases. Table 2 lists the leading databases that can help in characterizing the metabolites in three ways. For instance, these databases can (1) find the association of the metabolites to the host’s bio functions, (2) find the association of the metabolites to microbial functions, and (3) facilitate systems integration to link the host and microbiota via metabolite-enriched bio networks and pathways. Of these three types of databases, the host-specific database is possibly at its most mature phase. Part of the reason is that we have yet to fully comprehend the taxonomic determinants of the entire microbial community (Zhu et al., 2019).

**Table 2:** Available computation tools for developing predictive functional models using metabolomics and functional metagenomics.

Name	Description
KEGG Pathway Database	This partially open-access database can generate a multi-omics functional database. KEGG COMPOUND is focused on the functional analysis of mammalian metabolites and other small molecules (Kanehisa et al., 2017). Taking advantage of KEGG’s automatic annotation servers for metagenome sequences, KEGG pathways construction can inform the functional characteristics of metagenome (Kanehisa et al., 2016). Website: https://www.genome.jp/kegg/pathway.html
Ingenuity Pathway Analysis (IPA)	A commercial tool for multi-omics functional analysis. A specific sub-tool is focused on functional analysis of mammalian metabolites and other small molecules. Website: https://analysis.ingenuity.com
IMPaLA	An open-access enrichment analysis tool focused on functional analysis of mammalian metabolites. This tool can integrate gene/protein candidates with metabolites. Website: http://impala.molgen.mpg.de
MIMOSA	An open access system-level multi-omics integration tool that can deliver functional analysis of metabolites from mammalian and bacterial kingdom (Noecker et al., 2016). Website: http://github.com/borenstein-lab/MIMOSA
BioTransformer	An open-access systems integrative platform focused on functional analysis of metabolites from mammalian and bacterial kingdoms. Website: https://biotransformer.ca
gutMGene	A curated database that can perform functional integration of host and bacteria based on the metabolite and small molecular information. Website: http://bio-annotation.cn/gutmgene
MelonnPan	A model-based predictive platform for functional analysis of bacterial metabolites. Website: https://huttenhower.sph.harvard.edu/melonnpan/
VirHostNet	A model-based characterization of the functional network to inform the virus-virus interaction and virus-host interactions (Guirimand et al., 2015). Website: http://virhostnet.prabi.fr
HUMAnN3	An open-source analytical pipeline for functional analysis of mammalian and bacterial communities. As per the website, the functional analysis tends to answer the following question: “What are the microbes in my community of interest doing (or capable of doing)?” Website: https://github.com/biobakery/humann
MetaCyc	A curated database of metabolic pathways that take place across multiple kingdoms. At present, this database documents metabolic networks associated with bacteria, archaea, and several eukarya, such as fungi, etc. (Caspi et al., 2020). Website: https://metacyc.org/
Metage2Metabo (M2M)	A graph-based exploratory pipeline of annotated genome and metabolite to deliver functional analysis. Website: https://metage2metabo.readthedocs.io/en/latest/
MetExplore	An archive of curated and annotated metabolic networks in a collaborative environment (Cottret et al., 2018). Website: https://metexplore.toulouse.inra.fr/metexplore2/
AGORA	A correlative assembly tool to form the gut organisms through reconstruction and analysis; furthermore, a host-microbiome interactive network could be mapped by integrating the data with the Recon package (Magnúsdóttir et al., 2017). Website: https://github.com/opencobra/cobratoolbox
MaAsLin2	Meta-integration approach to data-driven modeling of microbial profile encompassing taxonomic, functional, or metabolomic features to generate metagenomic functional profile (Mallick et al., 2021) Website: https://github.com/biobakery/maaslin2_benchmark

This list excludes those that document only the omics/taxonomic information, such as locations of structural and functional regions, gene annotations, phylogenetic classification, and annotations, without presenting a tool to connect them to build functional networks to explain biomechanisms. A discussion about the open-source modules focused on similar network-building objectives could be consulted elsewhere.

There are mounting efforts to comprehend the biological functions of these metabolites and integrate them across various kingdoms (mammals, bacteria, etc.) and viruses based on their functional and biological relationships. These relationships are typically deduced either by statistical methods (e.g., correlative or enrichment analysis of the co-expressed metabolites) or by garnering the biochemical information (Amara et al., 2022) via curating the available literature that helps linking the metabolites to certain diseases (e.g., carcinoma) or biological networks (e.g., HPA axis abnormalities) based on *a priori* information. Subsequently, this information converges to build *ab initio* metabolite network topology (Naake and Fernie, 2019). For instance, the IPA and KEGG pipeline listed in Table 2 statistically integrate *a priori* knowledge to infer a multi-omics associative matrix (Subramanian et al., 2020) with metabolomics as one of the network layers. Next, this network topology could be mapped across the meta-organism to gain insight. Table 2 lists MIMOSA (Noecker et al., 2016), BioTransformer, and gutMGene (Cheng et al., 2022) databases that can inform about meta-organisms by integrating metabolomics across different kingdoms and organisms. This knowledge can lead us to design therapeutic strategies (Shi et al., 2021).

To summarize this chapter, metabolomics is a key sub-discipline of the pan-omics family (Brestoff and Artis, 2015; Bae et al., 2019; Krautkramer et al., 2021; Spivak et al., 2022), and microbial metabolomics is one of its impactful, though small in size components (Krautkramer et al., 2021). Ongoing efforts aim to link the metabolites to their upstream and downstream regulators that could be potential therapeutic targets (Olivotto et al., 1984; Lee and Finkel, 2013). This information-gathering process needs customization since the expression levels of the metabolites are susceptible to the host’s disease pathology, diet, geographical location, and circadian rhythm (Jones et al., 2021). To elaborate, the abundance of trimethylamine-oxide (TMAO), a liver-oxidized product of gut bacteria-derived TMA, is significantly over-expressed not only by certain disease types, such as type-2 diabetes and hepatic/renal diseases but also among the population who consumes fish-enriched diet (Subramaniam and Fletcher, 2018). Further, the sensitivity of metabolite profile to geographic location is underlined by a cross-continent diversification of breast milk-induced metabolite and microbiota (Gómez-Gallego et al., 2018). The compositions and characteristics of fecal microbiota and corresponding metabolites are highly susceptible to many indigenous and exogenous factors. Hence, the accuracy and reproducibility of detecting fecal metabolites critically depend on the study design and sampling protocol (Weinstock, 2012; Scherz et al., 2022). Potentially optimized protocols for fecal sample collection are discussed here (Mathay et al., 2015; Vogtmann et al., 2017; Jones et al., 2021). It is possibly essential to longitudinally collect fecal samples (Jones et al., 2021; Zheng et al., 2022) since it is unlikely that a single sampling of fecal materials can accurately represent the dynamic nature of microbiota.

All these factors should be considered to understand the *true* impacts of metabolites. In conclusion, systems knowledge integration could be the key to elucidating the relationship dynamics among different kingdoms, ecosystems, and the longitudinal profile of functional microbiota. The following chapters illuminate how metabolites play key roles in determining functional metagenomics—the bidirectional relationship between the microbiota and the host.

## Microbial ecosystem: diversity profile and disease pathogenesis

The hologenome encompasses nearly 1,600 times more microbial genes than the host genome (Lloyd-Price et al., 2016). This estimation alone can underscore the inherent complexity of meta-organism, which thrives on a symbiotic bidirectional relationship between host and resident microbiota. This host-microbiota crosstalk is shaped by and contributed to the host and its resident microbe’s coevolution, synchronized interactions with foreign elements, commensalistic association, and ecological or mutualistic collaboration (Ogunrinola et al., 2020); and its cumulative impacts are manifested in the microbial diversity profile (Manor et al., 2020). Therefore, it is contemplated that the quantitation of microorganismal diversity could throw light on disease etiology.

The estimation of microbial diversity primarily depends on the following two factors: *richness* (measures the number of independent species) and *evenness* (quantifies the relative abundances of different species). Alpha diversity measures the evenness and richness of microbial profiles within a community (Baczkowski et al., 1998). The routines, namely the Shannon diversity, Simpson diversity, and Chao 1 quantify different features of alpha diversity (GitHub, n.d.). Shannon diversity estimates the effective number of species colonized in a particular community, hence quantifying both evenness and richness (with a weight to evenness) of the microbial profile. Chao 1 estimates the number of species or the total richness of a particular community. Simpson diversity is primarily a dominance index, as this estimation gives more weight to the common or dominant species. On the other hand, beta diversity is the characteristic of the trans-community microbial profile (Baczkowski et al., 1998). The unweighted Unifrac quantifies the presence or absence of different taxa across the communities, whereas the weighted Unifrac considers the abundance of different taxa. The Bray–Curtis index estimates the abundance-based dissimilarity across the communities, while the Jaccard index measures the occurrence (presence vs. absence)-based diversity across the communities (GitHub, n.d.).

A shift from a balanced ecosystem or the dysbiotic ecosystem could be attributed to diet and lifestyle alterations (Ghosh et al., 2013), age (Mariat et al., 2009), obesity (Magne et al., 2020), circadian rhythm (Thaiss et al., 2014, 2015), and disease pathologies, including cancer (Sheflin et al., 2014; Biragyn and Ferrucci, 2018), cardiovascular disorder (Lau et al., 2017), immune dysfunction (de Oliveira et al., 2021), and several psychological illnesses (Sarkar et al., 2018; Parker et al., 2020). For instance, a reduced alpha diversity of gut microbiota was found in young adults (mean age: ~13 years) with attention-deficit hyperactivity disorder (ADHD). The measurement was estimated by Shannon diversity and Chao 1 index, which likely indicated a diminished richness of gut microbe linked to ADHD (Prehn-Kristensen et al., 2018). A contrasting picture emerged from an independent younger (mean age ~8 years) cohort, where Shannon diversity and Chao 1 index of subjects with ADHD emerged higher than that of the healthy baseline (Wang L. J. et al., 2020). These studies highlighted how multiple factors concurrently influence the microbial diversity.

Often, a combinatory analysis of alpha and beta diversity metrics is used to characterize the holistic changes in the microbial ecosystem. For instance, the alpha diversity in the fecal microbiota of cervical cancer patients showed no differences. Still, the beta diversity measured by the weighted Unifrac and Bray–Curtis algorithm revealed a significant difference that underscored a shift in trans-community microbial abundance, but not within a community (Wang Z. et al., 2019). Similarly, physiological stress caused significant alpha and beta diversity in gut microbes among young men. The Shannon dissimilarity and Chao 1 index suggested a shift in microbial richness within a community, while the Bray–Curtis analysis suggested a trans-community shift (Karl et al., 2017).

With the advancement of high-resolution detection technologies, we can now probe individual community members of the gut commensals. The abundance profile of a single microbe or its associative abundance profile with neighboring commensal(s) can deliver highly precise information. For instance, the shifting ratio of *Bacteroidetes* and *Firmicutes* has been linked to age (Mariat et al., 2009), obesity (Magne et al., 2020), and so forth. Linking the microbial abundance profile with circadian rhythm, the relative abundance of *Lactobacillus* was reported to escalate during the resting phase than during the active phase (Thaiss et al., 2014).

There is a growing appreciation for using the microbial diversity profile to develop the next generation intervention strategy. The torchbearer of the success story is the fecal microbiota transplantation (FMT) method that stalled tumor growth (Riquelme et al., 2019), ameliorated cardiovascular illness (Hu et al., 2019), and eliminated pathogenic insults (Hui et al., 2019) by maneuvering the microbial diversity. However, the potential of FMT as a treatment option is possibly limited due to the concerns about this intervention method’s traceability, safety, and standardization process (Osman et al., 2022; Vaughn et al., 2023). Driven by the hypothesis that the hypoxic condition inside the tumor is favorable for anaerobic microorganisms, systematic colonization of anaerobic bacteria successfully arrested the growth and metastasis of tumor cells (Drozdz et al., 2020). Further, the knowledge of dysbiosis helped to customize the diet supplements for immunotherapy to treat carcinoma (Routy et al., 2018).

Systematic manipulation of microbial colonies has emerged as a potential therapeutic option to combat several ailments; nevertheless, a comprehensive understanding of its molecular underpinnings is warranted to make this intervention process robust and effective. In this context, the next chapter highlights the microbial metabolites, which could play a critical role in designing a therapeutic strategy based on microbes. Once integrated with host metabolites, this knowledge could illuminate the biological underpinnings of their symbiotic relationship and lead to novel therapeutic options.

## Microbial metabolites and their spectrum of bio functions

In a homeostatic condition, the expression level of microbial metabolites is controlled by the host’s genetic predisposition, age (Connell et al., 2022), and other environmental factors, such as geographical location, food habits, and various lifestyle traits (Gupta et al., 2020; Krautkramer et al., 2021). Interestingly, there are less than two thousand microbial-specific metabolites in human compared to more than two hundred thousand host metabolites. However, the size of the microbial genome far outnumbers the human genome (Lloyd-Price et al., 2016; Metabolite Statistics, n.d.). A small number of microbial metabolites is potentially attributed to a rather streamlined metabolic function performed by the microorganisms. Ahmed et al. (2022) and Krautkramer et al. (2021) extensively reviewed these microbial-derived metabolites and reported how their biological functions reach various peripheral tissues to ensure the host’s health and fitness.

**Figure 2:**
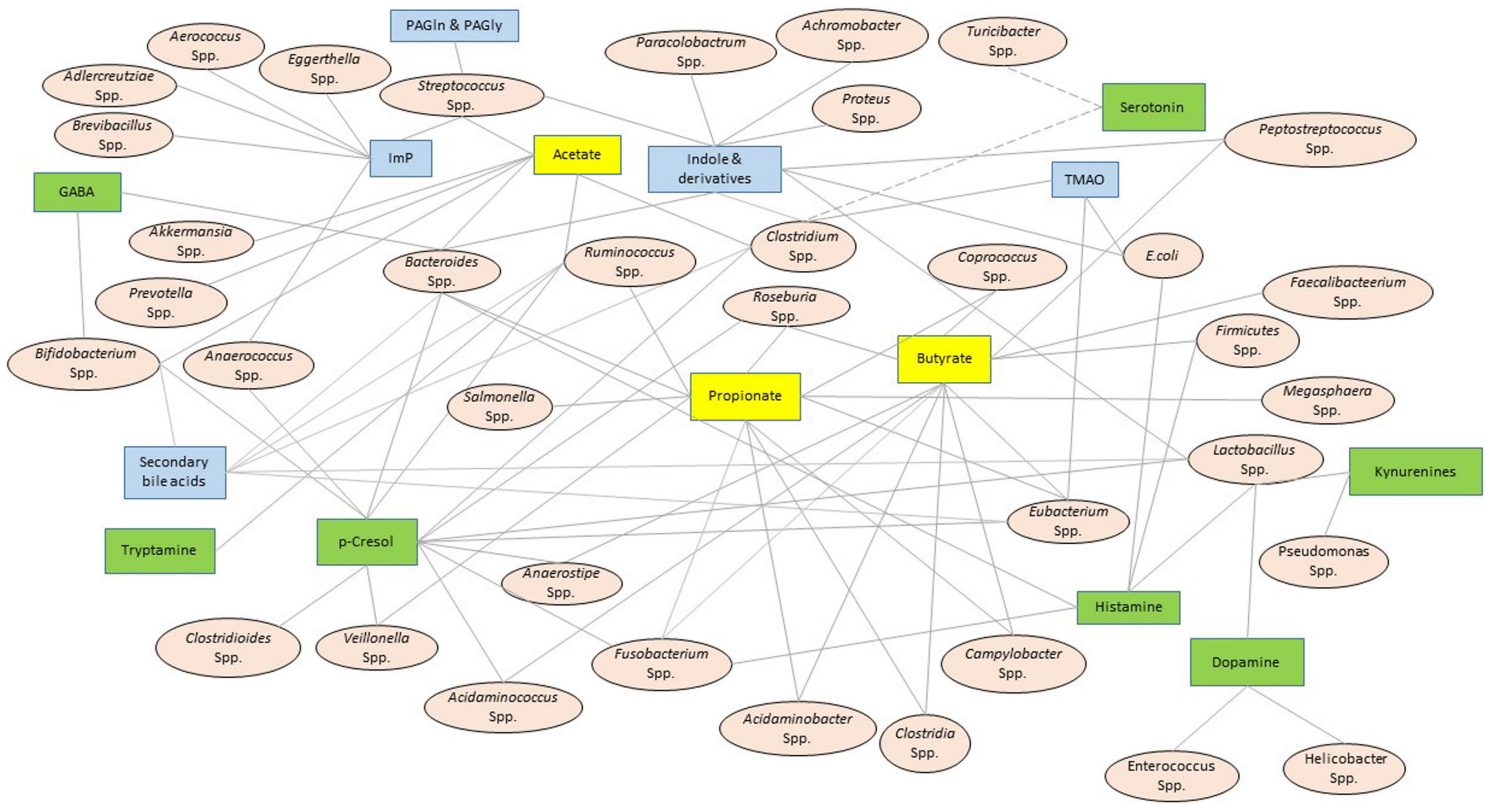
Bacteria-metabolite relationship network. The oval and square-shaped nodes represent the resident bacterial genera or species and the metabolite families, respectively. The solid edges connect the bacterial species or genera to their secretary metabolites. A set of broken edges that converge to serotonin connect it to those bacterial species that demonstrated correlative shifts in abundance, although there is no direct evidence of serotonin production from these bacteria (Yano et al., 2015; Fung et al., 2019). A double solid line connected GABA to *Bacteroidetes* to suggest that *Bacteroidetes* encodes an enzyme to produce GABA (Otaru et al., 2021). This network system was constructed after consulting a host of published literature (Pokusaeva et al., 2017; Rath et al., 2017; Wang et al., 2020; Krautkramer et al., 2021; Mou et al., 2021; Ahmed et al., 2022). The yellow, green, and blue boxes represent the metabolites linked to small chain fatty acids (SCFA), amino acids and their derivates, and others, respectively. GABA, γ-aminobutyric acid; TMA, trimethyl amine; Imp, imidazole propionate; PAGln, phenylacetylglutamine; PAGly, phenylacetylglycine.

Shifting microbial diversity from its eubiotic state is effectively mirrored by the microbial metabolite profile. Therefore, multiple ongoing efforts aim to manipulate the microbial metabolites to improve host’s health; however, these undertakings meet a significant challenge due to the lack of pertinent knowledge (Entwistle et al., 2019; Brunet et al., 2020). After screening several available literature (Yano et al., 2015; Rath et al., 2017; Fung et al., 2019; Wang S. P. et al., 2020; Krautkramer et al., 2021; Mou et al., 2021; Otaru et al., 2021; Ahmed et al., 2022), a bacteria-metabolite relationship network (Figure 2) was developed. This network informs the biological sites (e.g., bacteria) where a particular family of metabolites is synthesized. It is important to note that these networks are primarily built upon *a priori* knowledge; hence, the validity of such networks depends on continuous cross-checking of the literature and experimental feedback (Amara et al., 2022). Secondly, similar associative networks linking metabolites with other organisms, such as viruses and archaea, are essential to fully characterize the microbial metabolites’ functional outreach, and this aspect is yet underdeveloped. Table 2 presents the databases that aim to bridge this knowledge gap, and Figure 2 maps these microbial metabolites to their bacterial source.

Secondary bile acid (SBA) and short-chain fatty acids (SCFAs) are two major microbial metabolites in human. High-fat diets trigger the biosynthesis of SBA in the gut lumen (Ridlon et al., 2016; Zeng et al., 2019). Deoxycholic acid and lithocholic acid are two important SBAs produced by the dehydroxylation of primary bile acids (Ridlon et al., 2016; Zeng et al., 2019). In addition, gut microbiota epimerizes and oxidizes the primary and secondary bile acids to produce SBAs of iso-, alo-, oxo- and keto- families, which are comparatively low expressed entities, and their functional attributes are yet to be fully comprehended (Wahlstrom et al., 2016).

SCFAs are primarily comprised of acetate, propionate, and butyrate in an approximate molar ratio of 60:20:20, respectively, in a healthy host. Additional low-abundant SCFAs include fumarate, succinate, lactate, and pyruvate, which are used for cross-feeding among the different microbes (Macfarlane and Macfarlane, 2003; Silva et al., 2020). Anaerobic fermentation of non-digestible carbohydrates is the primary course of SCFAs production, as the pentose phosphate pathway and the Embden–Meyerhof–Parnas Glycolytic pathway catabolize five-carbon (e.g., xylene, pectins) and six-carbon (e.g., fructose, sucrose, starch, cellulose, etc.) substrates, respectively, to produce SCFA (Krautkramer et al., 2021). Briefly, the common end-product of the pentose phosphate pathway and Glycolytic pathway is phosphoenolpyruvate, which converts to pyruvate by endothermic cyclic conversion between NADPH and NAD+. Pyruvate is essentially the upstream substrate of all major SCFAs. For instance, the acetate and butyrate are produced from pyruvate via acetyl Co-A intermediate, with carbon dioxide and ethanol being the major byproducts, and propionate is generated via the succinate pathway utilizing carbon dioxide as the major co-factor (Fernandez-Veledo and Vendrell, 2019). Lactate and formate are additional downstream products from pyruvate metabolism. The production of these metabolites in microbes is typically controlled by access to dietary resources and stress factors. For instance, acetate and lactate are typically produced when carbohydrates are in excess; on the other hand, limited energy to the microbial community generally escalates the synthesis of propionate (Macfarlane and Macfarlane, 2003).

The scarcity of carbohydrates and/or high colonic pH induces the generation of SCFAs via protein fermentation (Neis et al., 2015). In addition to SCFAs, the protein fermentation in microbes produces folates, phenols, and indoles. Catabolism of branched-chain amino acids, such as leucine, isoleucine, and valine, produce isobutyrate and isovalerate, which are usually accumulated in low concentrations (Macfarlane and Macfarlane, 2003; Silva et al., 2020). Kynurenines and serotonin are the major products of the catabolism of tryptophan, an essential amino acid (Ostapiuk and Urbanska, 2022). Nearly 90% of the serotonin in the human body is produced by the colon, particularly the enterochromaffin cells on colonic epithelia. Currently, there is no direct evidence that microbiota produces serotonin, although such a possibility cannot be overruled since the microbiome encodes some contigs that typically contribute to the serotonergic network (Yano et al., 2015; Fung et al., 2019). In addition, a reduced concentration of serotonin was reported in concurrence with an increased abundance of tryptophan in germ-free mice, which indirectly suggests a microbial influence on serotonin production (Strasser et al., 2016). Another essential amino acid, namely histidine, undergoes decarboxylation in various bacterial species to produce histamine, and this recent discovery could have great potential in the field of allergic and immune therapy (Mou et al., 2021). Non-essential amino acids, such as tyrosine and L-dopa, and SCFAs, particularly butyrate, are metabolized in various bacterial species to produce dopamine, a key modulator of the gut-brain axis (Villageliu and Lyte, 2018).

Microbial metabolites could have beneficial or toxic roles depending on the metabolites’ expression levels and the target organ. For instance, low expression of SBAs (5–50 μM) promotes proliferation and invasiveness of colon cancer cells. Still, at higher expression levels (>50 μM), SBAs inhibit the colonic cell cycle and activate cancer cell apoptosis (Milovic et al., 2000; Amaral et al., 2009). Kynurenic acid operates differentially in different organs; in the CNS, kynurenic acid acts as a neuroprotective agent but inhibits insulin synthesis in the liver and kidney (Ostapiuk and Urbanska, 2022). SCFAs have various functions in bioenergy production, maintaining gut integrity, and promoting anti-inflammation via reactive oxygen species production (Tan et al., 2014). On the other hand, TMAO, a phosphatidylcholine derivative, is linked to oxidative stress, hyperlipidemia, and pro-inflammation (Agus et al., 2021).

**Figure 3:**
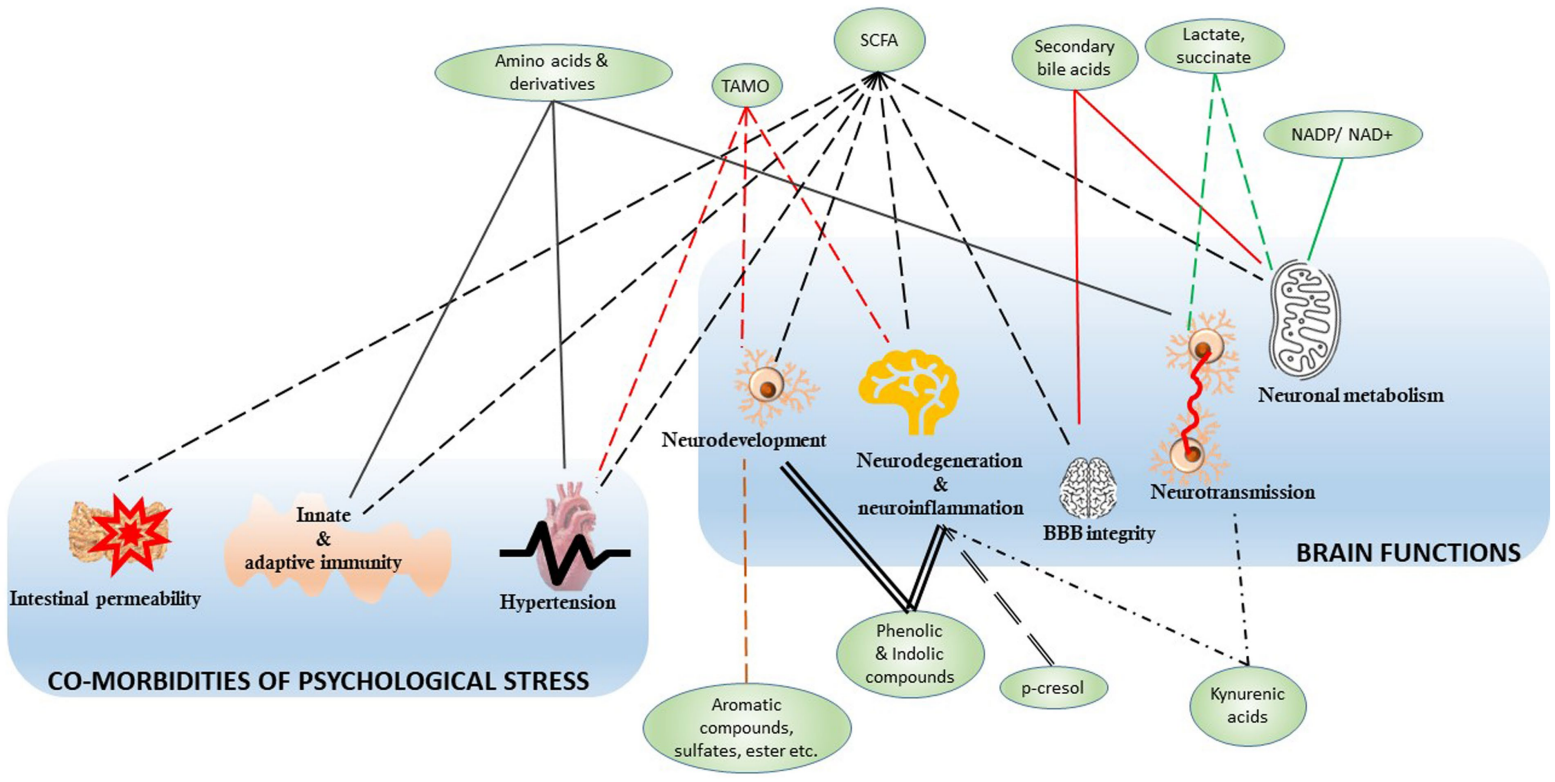
The microbial metabolites and associated brain functions and co-morbidities. The green nodes represent the microbial metabolites mapped in Figure 2 to associate with their generators, like the bacterial genera and species. The edges represent the connection between the metabolite and various functions, and all the edges were crafted differently to highlight their distinct connective modalities. The functions are clustered into two groups, namely, the brain functions and the co-morbidities of psychological stress. SCFA, small chain fatty acids; TMAO, trimethylamine N-oxide; NADP, nicotinamide adenine dinucleotide phosphate; NAD^+^, nicotinamide adenine dinucleotide.

In this context, there is growing traction about how gut microbiota communicate with peripheral organs. For instance, the dysbiosis of gut commensal has been linked to pulmonary health and asthma (Hufnagl et al., 2020), liver immunology (Trebicka et al., 2021), kidney failure (Zaky et al., 2021), and multiple cancer pathogenesis (Alexander and Turnbaugh, 2020; Sánchez-Alcoholado et al., 2020; Zheng et al., 2020; Kandalai et al., 2023; Xia et al., 2023). Indeed, an *in vivo* model suggested how microbial metabolites are associated with circadian rhythm and its disruption (Tahara et al., 2018). The roles of microbial metabolites in bioenergy production, somatic inflammation (Alexander and Turnbaugh, 2020), and physiological performances (Borrego-Ruiz and Borrego, 2024) have been studied extensively. Because psychological issues have become a prevalent health concern in the modern world, the study on the gut-brain axis is at the epicenter of the field of host-microbiome study. Figure 3 illustrates the functional association of these microbial metabolites to various brain diseases and co-morbidities, and the next chapter is focused on the gut microbiome-metabolite-brain axis.

## Gut microbiota-metabolite-brain axis

The gut microbiota-brain axis is a feedback-controlled circuit that recruits metabolites as the primary messenger between the gut and brain (Mayer et al., 2015; Ahmed et al., 2022). Due to the limited access to the human brain, the concept of the gut-brain axis has thus far been primarily built upon animal models. Mounting evidence shows that the gut microbiota-metabolite-brain axis controls the neurophysiology and signaling networks and regulates a spectrum of behavioral constructs. The association between the resident commensals and cerebral health was suggested by interrogating a colony of germ-free mice that demonstrated inhibited expression of tight junction proteins on BBB coupled with escalated BBB permeability (Braniste et al., 2014; Parker et al., 2020). Similarly, longitudinal antibiotic treatment reduced the abundance of gut commensals in mice, and this cohort demonstrated a cognitive deficiency and memory loss, as well as diminished neurogenesis in the hippocampus, the primary brain region to process memory (Fröhlich et al., 2016). Increased activity of a major component of the sympathetic nervous system in germ-free mice, namely the hypothalamic-pituitary-adrenal (HPA) axis, potentially orchestrated elevated Bdnf gene expressions (Manca et al., 2020) and accumulation of corticosterone in the bloodstream (Neufeld et al., 2011). Linking the gut commensal to social skill development, Desbonnet et al. (2014) reported that the germ-free mice showed increased social avoidance and self-grooming along with decreased social engagement. This negative outcome was reversed by administrating probiotic diets that helped growing the commensals in the host (Desbonnet et al., 2014; Fröhlich et al., 2016).

Differential microbial signatures, such as a shift in the diversity of the entire ecosystem (Uronis et al., 2011; Prehn-Kristensen et al., 2018; Tran et al., 2019; Wang L. J. et al., 2020) or a change in the comparative abundance of selected taxonomic groups (e.g., *Firmicutes* and *Bacteroidetes* ratio) (Mariat et al., 2009; Magne et al., 2020) are linked to various psychological deficiencies and co-morbidities. To elaborate, traumatic brain injury and post-traumatic stress disorder—the two most prevalent psychological disorders of modern warfare—are linked to fecal microbiota. For instance, a social stress mouse model stimulating PTSD-like traits caused a time-independent shift in the overall abundance profile of gut bacteria alongside a time-resolved alteration of the *Firmicutes* and *Bacteroidetes* ratio (Gautam et al., 2018; Hoke et al., 2022). Concurrently, these stressed mice displayed behavioral deficiencies, metabolic dysfunction, neurotoxicity (Muhie et al., 2017), and a shift in the neuronal morphology in the hippocampus, amygdala, and prefrontal cortex (Anilkumar et al., 2022). In fecal samples, the total abundance of bacterial phyla, namely *Actinobacteria*, *Lentisphaerae*, and *Verrucomicrobia,* was directly associated with the increased PTSD index (Hemmings et al., 2017). The proliferation of obligate anaerobic bacteria such as *Deferribacteres* in fecal samples of traumatic brain injury (TBI) patients potentially highlights that TBI triggers a hypoxic condition in the colonic lumen due to endogenous energy deprivation (Nicholson et al., 2019; Angoa-Pérez et al., 2020).

Gut-brain communication is facilitated by transferring neurotransmitters and neuromodulators via the bloodstream or lymphatic systems and synaptic transmission via the vagus nerve (VN). Most bloodborne microbial metabolites cross the BBB via carrier proteins and receptors expressed on BBB or by compromising BBB integrity (Ahmed et al., 2022). VN, a significant component of the parasympathetic nervous system, constitutes a parallel communication channel between the gut microbiota and the nervous system, encompassing the CNS and enteric nervous system (ENS) (Parker et al., 2020). *In vivo* studies observed that the microbial population was not directly connected to the VN because the nerve fibers do not penetrate the gut epithelia. Instead, the changes in the microbial ecosystem and corresponding shifts in metabolite levels perturb the neuroendocrine and endocrine signals of the host gastrointestinal tract; downstream signal travels to the brain via a series of receptors on the vagal afferents and neuropod cell-mediated synaptic transmission (Parker et al., 2020; Ahmed et al., 2022).

The intestinal epithelia play an integral role in the communication between brain and gut microbiota. Perturbed by the stress and environmental shifts, the neurons, immune cells, and mucosal cells of the intestinal epithelia release catecholamines, serotonin, dynorphin, and cytokines into the gut lumen. Playing a critical role in this process, the enterochromaffin cells in intestinal epithelia potentially act as the first line of the recipient of information about food intake, as it rapidly transmits this knowledge to the brain via activating VNs [sometimes via a single synapse within a millisecond timeframe (Kaelberer et al., 2018)] and/or sending bloodborne messengers to the hypothalamus; henceforth these cells regulate food intake and glycemia (Bohórquez et al., 2015; Gribble and Reimann, 2016). This enterochromaffin cell-mediated serotonin production is modulated by microorganisms such as *Clostridium* spp. and their metabolites, such as indole, norepinephrine, and butyric acid (Yano et al., 2015; Fung et al., 2019). This serotonin pool, often called the peripheral serotonin (El-Merahbi et al., 2015) functions independently of the serotonin pool generated inside the CNS since serotonin cannot cross BBB (Berger et al., 2009). The role of brain serotonin in controlling mood, sleep, and stress response is comparatively well studied (Berger et al., 2009; Strasser et al., 2016), while we have not fully comprehended the role of peripheral serotonin (El-Merahbi et al., 2015). The SBAs are another major microbial metabolite that cannot cross the BBB, although preliminary indications suggest certain roles for SBAs in modulating the brain functions (Monteiro-Cardoso et al., 2021).

The stress signals released into the intestinal lumen via the gut epithelial cells alter the pH and viscosity of gastrointestinal fluid along with the intestinal lumen’s temperature and pressure (Parker et al., 2020; Ahmed et al., 2022). As a result, the microbial compositions and their motility adapt to best utilize the available resources, such as excess lactate or reduced bioenergy, to survive in unfavorable environments. If the changes in the gut microbial ecosystem exceed normal homeostatic ranges, a therapeutic intervention becomes necessary; furthermore, if these processes are left unchecked, the intestinal epithelia become permeable, allowing the gut microbiome to enter the bloodstream- this condition is known as gut leakage (Chakaroun et al., 2020). Given changes in the functional metagenomics is mirrored by the differentially expressed microbial metabolites.

The altered ecology of commensals differentially secrete a wide variety of neurotransmitters derived from aromatic amino acids, which act on the brain functions as excitatory (e.g., glutamate, dopamine, and acetylcholine) or inhibitory neurotransmitters (e.g., GABA and glycine) (Spivak et al., 2022). It is also important to note that the synthesis and biofunctions of these neurotransmitters are controlled by various co-factors (e.g., age) and multiple peripheral tissues (e.g., kidney and liver). Still, the degree of influences of these co-factors on the gut commensals remains largely unexplained.

Glutamate, the most prominent neurotransmitter in the cortex region of the brain, is regulated by kynurenic acid, and colonic *Lactobacillus* and *Pseudomonas* spp. contribute a significant portion of whole-body kynurenic acid accumulation via tryptophan metabolism (Ostapiuk and Urbanska, 2022). The synthesis of dopamine, another excitatory neurotransmitter, is controlled by p-cresol and synthesized in several peripheral tissues and colonic *Lactobacillus* and *Clostridium* spp. (Pascucci et al., 2020). In addition to modulating the dopaminergic network, p-cresol regulates the oxytocinergic and opioidergic networks and guides behavioral plasticity (Putnam and Chang, 1858). On the other hand, *Bacteroidetes*, one of the most abundant fecal microorganisms, encodes glutamate decarboxylase-encoding gene, which is linked to the production of GABA, the major inhibitory neurotransmitter of brain (Otaru et al., 2021).

*Clostridia*, *Fusobacterium*, and *Acidaminobacter* are the primary producers of SCFAs, while *Firmicutes*, a highly abundant fecal microorganism, mainly secretes butyrate (Eeckhaut et al., 2011). SCFAs, primarily propionate and butyrate, participate in many health-beneficial function (Louis and Flint, 2017); for instance, they control the adaptive immune systems (Louis and Flint, 2017) and, in this process, mitigate neuroinflammation by inhibiting the production of histone deacetylase 1, a proinflammatory cytokine (Song et al., 2022).

In a healthy gut milieu, lactate is used to cross-feed the microbiota for unrelenting SCFA production. In homeostatic conditions, many gut microbes, including *Firmicutes,* convert lactate to propionate and butyrate (Abedi and Hashemi, 2020), which helps maintain a low accumulation of lactate in the intestinal lumen (Duncan et al., 2004). Increased lactate production is typically pH mediated and often identified as the marker of dysbiosis when *Firmicutes* and *Bacteroidetes* get replaced by lactate-producing *Actinobacteria*, *Lactobacillus,* and *Proteobacteria*. This event inhibits the synthesis of butyrate and propionate (Abedi and Hashemi, 2020; Wang S. P. et al., 2020). Interestingly, increased lactate production by gut anaerobes during exercise potentially supports the host’s mitochondrial respiration (Brooks, 2020) and indirectly facilitates cerebral BDNF production via upregulating the Sirtuin1 network. This is likely a dose-dependent impact of lactate on brain, as the mice under the regime of physical exercise showed increased learning and memory retention power (El Hayek et al., 2019).

In addition to lactate, several other microbial metabolites actively participate in neuronal bioenergetics and glucose homeostasis (Zhang et al., 2021). Potentially directed by enterochromaffin cells, microorganisms inside the intestinal lumen produce glucose via gluconeogenesis (Soty et al., 2017), which switches to lactate production during energy deficiency and hypoxia (Brooks, 2020). Although the microbe-induced glucose metabolism has been comparatively well studied (van Olden et al., 2015; Utzschneider et al., 2016), some pertinent aspects still need additional probing. For instance, the energy-modulating hormones, such as peptide YY and GLP-1, are found to be expressed on intestinal epithelia, and corresponding receptors are reportedly expressed in the hypothalamus (Steinert et al., 2011), although the mode of exchange of these hormones between the gut and brains is yet obscured.

Finally, recent studies have suggested a potential association between the host microbial community and the glymphatic system (Hablitz and Nedergaard, 2021), a novel concept that explains the waste clearance pathways exclusively from the mammalian CNS (Meyerhoff et al., 2022). Glymphatic systems’ close associations with BBB and sleep construction, and therefore with the tryptophan pathway and vagal retrograde signaling network, intrigue us to link the gut microbiome to glymphatic systems (Hablitz and Nedergaard, 2021); however, much work is needed to completely understand this mechanism.

## Challenges and path forward

Systems interrogation and knowledge integration of functional metagenome, meta-organism, and host omics are gaining traction to uncover the holistic molecular mechanism that drives the host’s stress response. Most of the available data, particularly those focused on the gut-brain axis, are built upon animal models due to the limited availability of the human brain. However, the translational potential of rodent metagenomic data is often contested. The brain morphology of humans and rodents is fundamentally different since the human gyrecephalic brain has distinct convolutions and expansions in the cortex, which facilitate handling a much wider range of emotional structures than that by the smooth-surfaced cortex in the lissencephalic brain of rodent (Sun and Hevner, 2014). Furthermore, the ecosystem of rodent microorganisms is distant from that of human; conversely, domestic animals, such as dogs, have higher metagenomic homology with humans (Wardeh et al., 2015). On the positive side, the enterochromaffin cells of rodents were found genetically homologous to that of human, although their functional homology, particularly under stress, is yet to be fully characterized (Roberts et al., 2019). Some of the customized rodent models, such as the germ-free model and gnotobiotic humanized model, are promoted for greater translational potential (Uzbay, 2019). These models could reduce the high variability of metagenomic data among the research laboratories. Nevertheless, germ-free mice are predisposed to various unique characteristics due to their atypical habitat, limited maternal care, and distinct lifestyles and diets (Mayer et al., 2015). Altogether, these traits potentially limit the translational potential of the outcome derived from germ-free mouse colonies (Mayer et al., 2015).

The *true* characteristics of the host-gut microbe relationship are still obscure; to begin with, we are still unsure if this relationship is causative or correlative in nature. Comprehension of the host-microbe relationship possibly depends on the following two fundamental pieces of information: (i) how the gut microbe communicates with peripheral organs and (ii) how different microorganisms in the gut lumen, such as bacteria, viruses, etc. crosstalk among themselves. To shed light on the gut microbe-organ axis, this review article has discussed in detail the topic of the gut microbe-brain axis, while the other relationship matrix with gut microbes and other peripheral organs, such as lungs, liver, and kidneys, were cursorily discussed. The inter-organism relationship, particularly the bacteria-virus mutualistic association, has been considered to be the controlling factor of host defense. Enteric virus is mostly represented by bacteriophage and eukaryotic virus (Li et al., 2021), although the characterization of the virus composition has been challenged by limited capability in mapping the virus gene sequences (Minot et al., 2011; Manrique et al., 2016). Given that the bacteriophage can obliterate bacteria, their relationship and comparative abundance in the intestine remains an interesting subject to moot. A significantly high ratio of bacteriophage-to-bacteria at the enteric mucosal surface in comparison to the rest of gut lumen (Barr et al., 2013) is a potential frontline of defense against bacterial infection and thereby regulates many antagonists and beneficial actions including the host defense and immune response (Almeida et al., 2019; Kirsch et al., 2021). Our understanding of additional inter-kingdom relationships, such as bacteria-archaea (Hoegenauer et al., 2022) and bacteria-protozoa (Dubik et al., 2022), are still at their early stages, although emerging studies identified their concerted efforts in disease pathogenesis (Kodio et al., 2020; Mafra et al., 2022).

Needless to say, we have yet to fully characterize the associations between microbial metabolites and disease pathology. One of the possible modalities to meet the knowledge gap is to systematically dissociate a *sick* gut from its *sick* host. The null hypothesis could be that the fecal microbe of a *sick* host cannot adversely affect a *healthy* host. To support this hypothesis, fecal samples could be collected from the stressed or *sick* rodents and allowed to colonize them in healthy gnotobiotic rodents using FMT (Wang J. W. et al., 2019). Subsequent analysis can throw light on how a *sick* microbiota can control the host’s health in the absence of the adverse condition. Concurrent FMT of *healthy* commensals (i.e., the fecal samples collected from healthy cohort) into *sick* rodents will help in getting the full scenario about how microbiota communicate with the host to regulate its overall health.

Following the same concept, a surgical deletion or chemical manipulation of VNs can highlight the role of this nervous system in sensing the dietary intake (Brown et al., 2011; Yao et al., 2018) and consequent impacts on humans, such as the change in body weight (Burneo et al., 2002).

The outcome of the abovementioned modalities has been used to customize the traditional nutraceuticals, such as prebiotic, postbiotic, and symbiotic diets, and more novel supplements, namely parabiotic (made from non-viable microorganisms) and postbiotic biotherapies (made from microbial derivatives, such as metabolites) (Nataraj et al., 2020); although these nomenclatures are contested in past (Zolkiewicz et al., 2020; Salminen et al., 2021). Additional therapeutic approaches include genetic domestication of the microbe of interest using gene-editing technology (e.g., CRISPR) or by implanting synthetic promoters in the gut lumen (Mimee et al., 2016; Inda et al., 2019). In the recent past, *Bacteroidetes*, one of the most abundant gut commensals, was successfully systems-engineered (Mimee et al., 2016). It is a step toward designing a universally applicable tool that can systematically alter the microorganism along with its neighbors (e.g., *Firmicutes* that co-habitats *Bacteroidetes*) and/or co-factors (e.g., diet source) to reinforce the host’s response (Wexler, 2007; Inda et al., 2019).

Disease diagnostic and prognostic capabilities based on meta-organism data have shown significant progress. A sophisticated colonoscopy with a miniature camera can monitor a wide range of physiological attributes of the colon, such as pH, temperatures, etc., in real-time (Yung et al., 2016; Kalantar-Zadeh et al., 2017). Ingestible sensor prototypes, such as the “digital pill,” have emerged at the forefront to the disease diagnosis platforms (Kalantar-Zadeh et al., 2017; Beardslee et al., 2020). These advanced sensors can determine the colon oxidation potentials due to the shifts in microbial abundance (Baltsavias et al., 2020) and profile the gases, such as oxygen and hydrogen, emitted inside the colon lumen (Kalantar-Zadeh et al., 2018). Box 2 documents these novel diagnostic concepts along with the more conventional prototypes that monitor microbe and microbial metabolites.

Shifting the focus to its surrounding environment, metagenome and metabolites have shown great potential to enable 360° surveillance outreach. Wastewater surveillance of the microbiome gave a longitudinal profile of spreading COVID-19 infection across different communities (Brumfield et al., 2022). Metabolite monitoring in bryophytes and fish larvae (Sanches-Fernandes et al., 2022) can act as biosensors of toxins or radio-biological attacks on the communities.

Microbial metabolites are the primary intermediatory of the crosstalk between host and microbiota. This is an emerging concept essentially refining the traditional view of postprandial neuronal and hormonal exchange between the brain and gut (Wachsmuth et al., 2022). The microbiome is critically associated with the pathophysiology of several diseases and the host’s adaptive response to stress. Hence, one can anticipate that systematic modulation and monitoring of the microbiome could be the key enabler to combat diseases. Overall, meta-organisms and functional microbiota are the subject of very active research, and we hope to see significant progress in the coming years.

Box 1 Definitions of the key terms used in this article. The “*” items are those whose definitions are sometimes contested; see the main text for details.

**Table:** 

Term	Definition
Resident microbiota	Group of microorganisms, including bacteria, archaea, viruses, fungi, and protozoa that colonize in or on the host, such as human
Resident microbiome	Genome of the resident microbiota
Functional metagenome	Biological functions accomplished by the microbiota
Metabolites	Substrate, derivates, intermediatory agents, and end products of the bio functions
Metabolomics	Sub discipline of multi-omics that particularly deals with metabolites
Metabolite networks	Cluster of nodes that are connected via edges. Here, the nodes are represented by the metabolites, co-regulating diseases, and biological signals, and the edges are represented by functional and/or structural connections between the nodes
Microbial metabolites	The metabolites that are generated specifically from the biological actions undertaken by or in the microbiota
Hologenome	Combined assembly of gene and genome of resident microbiota and the host
Holobiont	A concept about studying the host-microbiota interactive commune that includes their combined biology, ecology, and evolution
Meta-organism	Bidirectional association encompassing the host and resident microbiota
Homeostasis	A balanced environment in an ecosystem that includes the host, microorganisms, and environment
Eubiosis	Balanced microbial ecosystem
Dysbiosis	Disbalanced condition of the microbial ecosystem, which is typically caused by disease onset, lifestyle change, or exposure to foreign agents/stressful conditions
Alpha diversity	The shift in the abundance profile of the microbial populations within a particular community
Beta diversity	The shift in the abundance profile of the microbial populations across the communities

Box 2 **An outlook of the conventional to futuristic tools/technologies that could readily benefit from the knowledge of meta-organisms and metabolites.**

**Table:** 

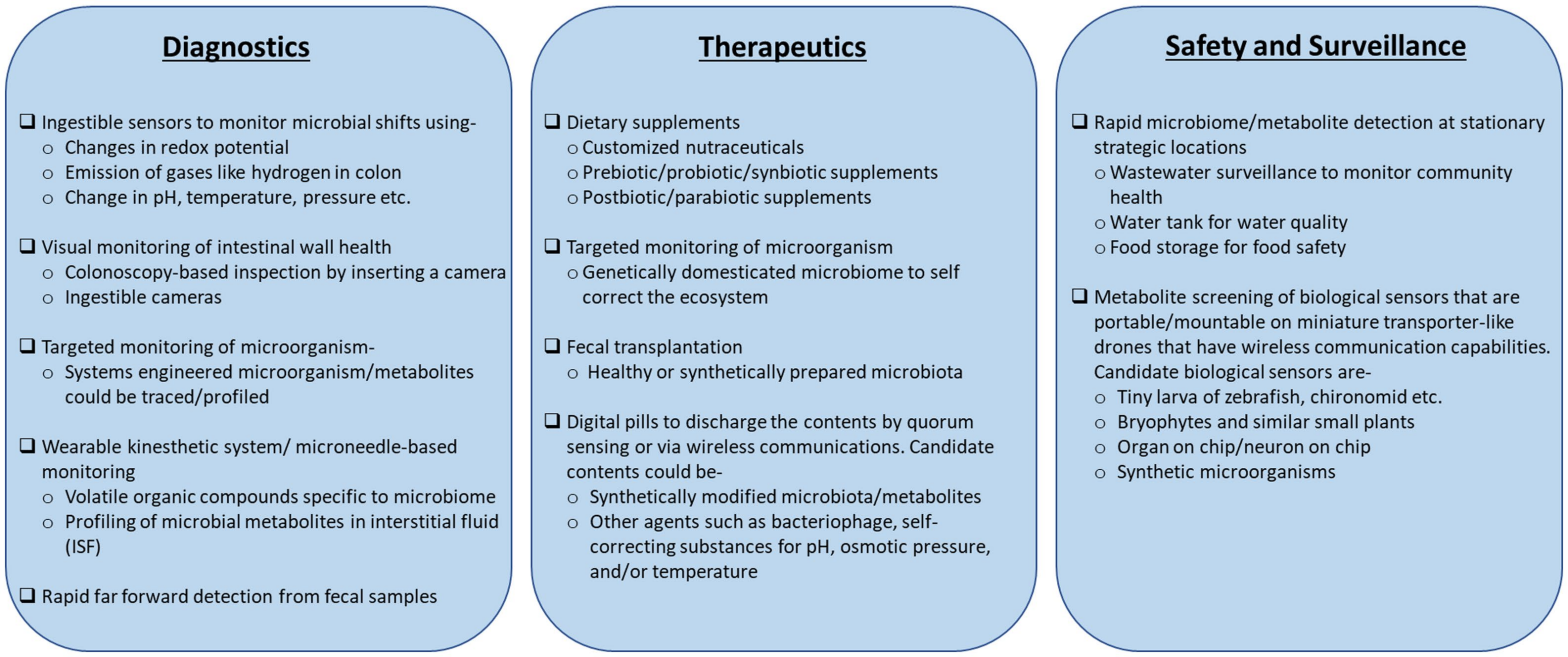

## Author contributions

NC: Conceptualization, Data curation, Investigation, Writing – original draft, Writing – review & editing.
